# Silencing circ‐USP1 protects the renal tubular from kidney injury induced by hypoxia via modulating miR‐194‐5p/DNMT3A axis in acute renal allografts

**DOI:** 10.1111/jcmm.16286

**Published:** 2021-01-23

**Authors:** Tao Yao, Dongqing Zha, Ping Gao, Xiaoyan Wu

**Affiliations:** ^1^ Department of Nephrology Zhongnan Hospital of Wuhan University Wuhan China

**Keywords:** acute renal allografts, circ‐USP1, DNMT3A, miR‐194‐5p

## Abstract

Recent studies indicate that circular RNAs are involved in dysregulation of kidney injury. Nevertheless, the underlying mechanisms remain largely unclear. Therefore, this study sought to investigate the role of circ‐USP1 in the pathogenesis of early renal allografts. Thirty‐two male C57BL/6J mice aged between 6 and 8 weeks were divided into the sham and allograft groups. Thereafter, the association between miR‐194‐5p, circ‐USP1 and DNMT3A was confirmed using a combination of bioinformatics and the luciferase reporter gene assay. Additionally, the expression of circ‐USP1, miR‐194‐5p and DNMT3A mRNA was detected through qPCR. Afterwards, the Western blot assay was performed to examine the expression of DNMT3A protein. Finally, the TUNEL assay was conducted to determine the rate of apoptosis in DNMT3A cells. The expression of circ‐USP1 increased, while that of miR‐194‐5p decreased in renal allografts. Additionally, silencing circ‐USP1 reduced kidney injuries caused by renal allografts in mice. Moreover, miR‐194‐5p was a target for circ‐USP1, and DNMT3A was a target of miR‐194‐5p. Finally, it was shown that silencing circ‐USP1 reduced DNMT3A expression in the kidney of mice that received renal allografts. Circ‐USP1 functions as a competing endogenous RNA for miR‐194‐5p. This occurs in order to regulate DNMT3A expression in kidney injury induced by hypoxia in acute renal allografts.

## 1 INTRODUCTION

Kidney transplantation is an important intervention for patients with end‐stage renal failure. The procedure is able to effectively prolong survival time and improves patients’ quality of life.^1^ Although the graft survival rate, 1 year after kidney transplantation is on the rise, the long‐term rate of survival (10 years) has stagnated in about 10 years.^2^ Moreover, the application of more effective immunosuppressive agents has greatly reduced the number of patients with acute rejection (AR) after kidney transplantation in the past few decades. However, the lack of non‐invasive molecular markers has frustrated efforts to optimize ‐rejection treatment options.^3^ Presently, renal biopsy is the ‘gold standard’ for the detection of dysfunctional kidneys after a transplant.^3^ Nonetheless, the technique has certain limitations including high cost, it is invasive, causes multiple complications and sampling errors may occur.^3^ Renal biopsy may however completely and accurately reflect the changes in renal function.^3^ Notably, AR poses a significant risk of chronic rejection (CR),^4^ yet the prognosis of CR is worse than that of AR.^5^ Therefore, it is necessary to find non‐invasive markers that will aid in early and effective diagnosis of AR. This will help in preventing the occurrence of AR after kidney transplantation and provide personalized treatment options for patients.

Circular RNAs (circRNAs) represent a class of non‐coding RNAs that are extensively expressed in mammals.^6^ Recent studies show that circRNA can act as a sponge that adsorbs microRNAs (miRNAs) and perform biological functions. Additionally, they may be involved in regulating transcription and post‐transcriptional processes, alternative splicing, encoding proteins, and acting as protein decoys.^7^, ^8^, ^9^ Research evidence also indicates that they are detectable in the blood of patients who suffered from acute kidney injury.^10^ Moreover, it was shown that urinary hsa_circ_0001334 was a new biomarker of AR.^10^

On the other hand, miRNAs are endogenously produced as single‐stranded small RNAs of about 22 nucleotides. They bind to the 3' non‐coding region (3' UTR) of the target gene through their seed sequence, leading to degradation or inhibition of post‐transcriptional translation.^11^ miRNAs serve as vital regulatory roles in physiological processes including cell differentiation, proliferation and apoptosis.^12^ Extensive research indicates that miRNAs are closely associated with the occurrence of AR.^13^, ^14^, ^15^ Moreover, recent evidence shows that miR‐194‐5p is involved in the progression of various diseases including acute myeloid leukaemia, clear cell kidney carcinoma and hepatocellular carcinoma.^16^, ^17^, ^18^ A previous study showed that miR‐194‐5p was a target of circ‐USP1 and both were involved in regulating the permeability of the blood‐tumour barrier.^19^ Nevertheless, to the best of our knowledge, the role of miR‐194‐5p and circ‐USP1 in the progression of kidney injury induced by hypoxia in acute renal allografts remains unknown.

DNMT3A is a 130‐kDa protein that is highly conserved in vertebrates and shows 98% homology between humans and mice.^20^ Previous research indicates that circ‐DNMT3A was down‐regulated in mice with acute kidney injury.^21^ However, slight attention has been paid to the potential role of DNMT3A in kidney injury induced by hypoxia in acute renal allografts.

In this study, the expression and role of circ‐USP1, miR‐194‐5p and DNMT3A in kidney injury induced by hypoxia in acute renal allografts were investigated. The results showed that the expression of circ‐USP1 was elevated in renal allografts in a time‐dependent manner. Notably, the expression of miR‐194‐5p, a previously reported circ‐USP1 target,^19^ decreased in diseased kidneys. Additionally, silencing circ‐USP1 in mice protected them from kidney injury following a renal allograft. Finally, reduced expression of circ‐USP1 was accompanied by increased miR‐194‐5p and decreased DNMT3A expression in kidneys.

## 2 METHODS

### 2.1 Animal grouping and kidney transplantation

Thirty‐two male C57BL/6J mice aged between 6 and 8 weeks were purchased from Wuhan University. The mice were then divided into two: the sham and allograft groups. The sham group was injected with 2% sodium pentobarbital (40 mg/kg) after anaesthesia; then the bilateral renal pedicles were exposed through an abdominal incision. In the allograft group, the renal pedicle was clamped with a non‐injured arterial clip for 25 minutes. The renal pedicle was then opened for perfusion.^22^ After ischaemia, the kidney appeared uniformly purple‐red, but the purple pigmentation disappeared after removing the arterial clip. The ischaemia‐reperfusion was deemed successful upon this observation. In the control group, the free kidney pedicles were opened without clipping. After 25 minutes, the abdominal cavity of each group was sutured layer by layer. During the operation, the mice were placed on a thermostatic electric blanket and maintained at 37°C. After anaesthesia, they were awakened and proof of life was established. After survival was confirmed, the mice were returned to the cage.

To evaluate the role of circ‐USP1 in injuries caused by kidney transplantation, si‐circ‐USP1 or si‐control (3 mg/kg body weight) formulated with lipid nanoparticles (Lipofectamine 3000) was intravenously injected into mice before allograft surgery. The renal allograft mouse models were established one day after injection. This study was approved by the Ethics Committee of the Animal Research Institute of Zhongnan Hospital of Wuhan University.

### 2.2 Sample collection

After successful modelling, the mice were anesthetized according to the perfusion time of 0.5d, 1d and 3d after which blood and kidney specimens were collected. The collected blood was then centrifuged at 660 *g* for 15 minutes. Following centrifugation, the upper serum was collected and preserved in a refrigerator at −80°C. The level of serum creatinine was measured utilizing the Olympus analyzer. To obtain a kidney sample, the inferior vena cava was isolated and 0.9% sodium chloride solution infused through the left ventricle. Afterwards, the kidney was removed once it turned white then 1/3 of it fixed in 4% tissue cell fixative for morphological observation. The remaining portion of the kidney tissue was saved in liquid nitrogen for subsequent detection of mRNA and protein. Specimens for the control group were collected at the same time points indicated above.

### 2.3 Morphological observation of kidney tissue

After fixing the kidney tissue with 4% tissue cell fixative solution for 24 hours, it was dehydrated, embedded in paraffin, sectioned (3 μm), and subjected to HE staining. Afterwards, measurement and scoring of renal tubulointerstitial pathological damage were blinded and completed by 2 experienced nephrologists. To observe and score renal pathological damage, each slice was randomly selected under a 200‐fold light microscope from 10 non‐overlapping visual fields at the junction of the cortex and medulla. The scores were as follows: a tubule scored 0 points, a damaged renal tubule interstitial area <25% scored 1 point, 25% ~ 50% scored 2 points, 50% ~ 75% scored 3 points and >75% scored 4 points. This was a semi‐quantitative analysis that calculated the average values. The higher the score, the more severe the damage was.^23^

### 2.4 Cell culture, transfection and model construction

Immortalized human renal proximal tubule (HK‐2) cells were purchased from the American Type Culture Collection (Manassas, VA). The cells were then cultured in DMEM medium consist of 10% FBS with 100 μg/mL penicillin and 100 µ/mL streptomycin at 5% CO_2_ and 37°C. The culture medium was replaced every 2‐3 days. In order to establish an in vitro ischaemia/reoxygenation model, cells were cultured in a hypoxic environment with 1% O_2_, 94% N_2_ and 5% CO_2_ for 3 hours. Then they were fostered in a complete medium with 21% O_2_ for different periods (1, 3, 6, 12 and 24 hours).

### 2.5 qPCR for detection of miR‐194‐5p, circ‐USP1 and DNMT3A mRNA levels

The tissues/cells were lysed with TRIzol, extracted with chloroform and isopropanol, centrifuged, and rinsed twice with 70% ethanol. Following this, the ethanol was evaporated at room temperature for 20 minutes after which DEPC water was added to extract total RNA. Afterwards, the PrimeScript™ RT Master Mix (Takara, China) was utilized to reverse‐transcribe RNA to cDNA as the manufacturer's instructions. Finally, the levels of miR‐194‐5p, circ‐USP1 and DNMT3A mRNA were measured using TB Green® Premix Ex Taq™ (Takara, China) according to the manufactures directions. β‐actin was utilized as an internal control. The experimental statistics was then evaluated through the 2^−ΔΔCt^ approach. The primers used are listed in Table S1.

### 2.6 Western blot detection of DNMT3A protein expression

Briefly, the kidney tissues and cells were lysed with the RIPA lysis buffer, placed on ice for 30 minutes, and then ultrasonically disintegrated. The supernatant was collected via centrifugation at 2640 *g* for 15 minutes at 4°C. The concentration of each histone was balanced then pre‐warmed 5× loading buffer added to each. Afterwards, a 37°C water bath was used for 30 minutes to detect β‐actin. Additionally, the water was boiled at 100°C for 5 minutes in order to detect the DNMT3A protein. 30 μg of protein sample was then added to each well for 10% polyacrylamide gel electrophoresis at a concentration gel voltage of 80 mV and the separation gel voltage of 120 mV. Thereafter, the primary (polyclonal rabbit anti‐DNMT3A antibody 1:1000; polyclonal rabbit anti‐GAPDH antibody 1:1000, incubated overnight at 4°C) and secondary antibodies were incubated (HRP‐labelled goat anti‐rabbit secondary antibody 1:7000, incubated on a horizontal shaker for 60 minutes at room temperature). ECL was used to develop colour. Finally, the gel image processing system was performed to quantitatively analyse the target band.

### 2.7 TUNEL assay to determine the rate of apoptosis in DNMT3A cells

The TUNEL reaction solution (purchased from Beijing Zhongshan Biological Company) was added dropwise to each group of cell suspension after trypsin digestion. Thereafter, the eggs were incubated for 2 hours at 37°C, washed 3 times with PBS then peroxidase‐labelled anti‐ground added dropwise. This was followed by an incubation of the eggs and the Gauxin antibody in a humid box at 37°C for 25 minutes. DAB was used for staining and haematoxylin utilized as a counterstain. Furthermore, PBS was used instead of the TUNEL reaction solution as a negative control. The positive control section was pre‐treated with DNase I for 8 minutes and then stained based on the TUNEL method. The nuclei of apoptotic cells were brown, and 5 fields of view were observed in each section. Moreover, 200 cells were calculated in each field and then the rate of apoptotic cells was measured.

### 2.8 Luciferase reporter assay

The pmirGLO‐DNMT3A‐ wild type (WT), pmirGLO‐DNMT3A‐ mutant (MUT), pmirGLO‐circ‐USP1‐WT, pmirGLO‐circ‐USP1‐MUT and miR‐194‐5p mimics were obtained from were purchased from Sangon (Shanghai, China). To verify the correlation between circ‐USP1 and miR‐194‐5p, we utilized Lipofectamine^®^ 3000 to co‐transfect the constructed WT‐circ‐USP1 or MUT‐circ‐USP1 and mimic miR‐194‐5p mimic or its negative control into the HK‐2 cells. Additionally, to validate the interaction between DNMT3A and miR‐194‐5p, we applied Lipofectamine^®^ 3000 to co‐transfect the constructed WT‐ DNMT3A or MUT‐ DNMT3A, and mimic miR‐194‐5p mimic or its negative control into the HK‐2 cells. After transfection, cells were subjected to the dual‐luciferase reporter assay based on the manufacturer's protocol. The relative luciferase activity was normalized to renilla luciferase activity.

### 2.9 Statistical analysis

The SPSS 25.0 software was utilized for statistical analysis. Measurement data were expressed as means ± SD. Additionally, one‐way ANOVA was performed for comparison between multiple groups, and the LSD test was employed for pairwise comparison between categories. Finally, the Dunnett T3 test was used in cases of uneven variance. *P* < .05 was considered to be statistically significant.

## 3 RESULTS

### 3.1 The expression of circ‐USP1 elevated and miR‐194‐5p reduced in renal allografts

**FIGURE 1:**
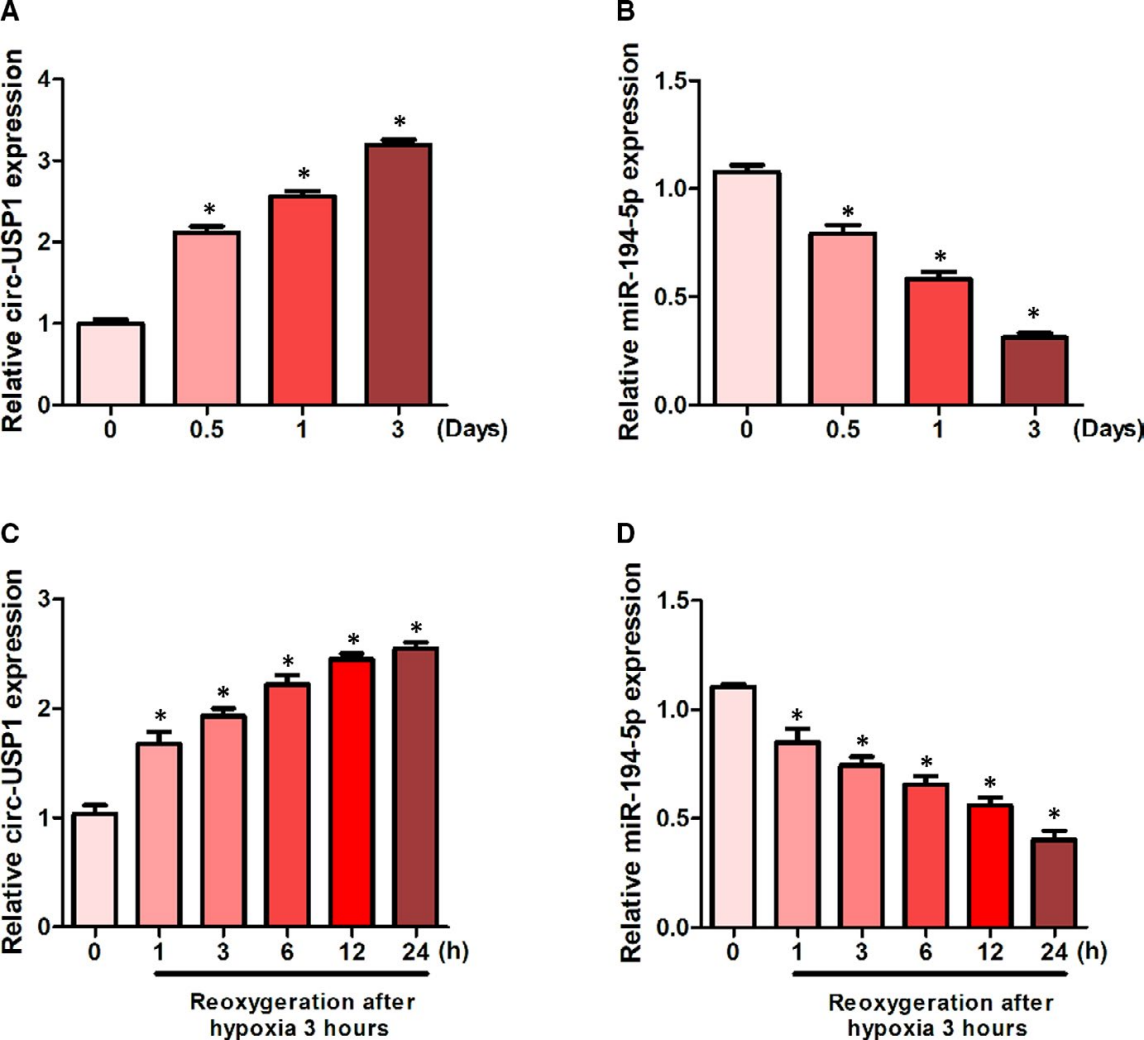
The expression of circ‐USP1 elevated and miR‐194‐5p reduced in renal allografts. (A, B) Relative expression of circ‐USP1 (A) and miR‐194‐5p (B) in the kidney of mice that received renal allografts at different time points. (C, D) The expression of circ‐USP1 (C) and miR‐194‐5p (D) in HK‐2 cells at different time points. *P < .05

To investigate abnormal gene expression in response to ischaemia in vivo and in vitro, renal allografts at early rejection were observed in engrafted kidneys. The results of RT‐PCR indicated that circ‐USP1 significantly increased in a time‐dependent manner at day 0.5, day 1 and day 3 post‐engraftment. In contrast, miR‐194‐5p remarkably decreased in a time‐dependent fashion, in the kidney of mice that received renal allografts compared with the group at day 0 (Figure 1A,B). Additionally, the HK‐2 cells were cultivated in vitro, in an ischaemic condition (1% O_2_), followed by 1, 3, 6, 12 and 24h reoxygenation to mimic hypoxia/reoxygenation (H/R). Compared with the group on day 0, the circ‐USP1 expression was significantly elevated in a time‐dependent manner. However, the expression of miR‐194‐5p remarkably reduced in a time‐dependent fashion in vitro (Figure 1C,D).

### 3.2 Silencing circ‐USP1 reduced injury in the kidney of mice that received renal allografts

**FIGURE 2:**
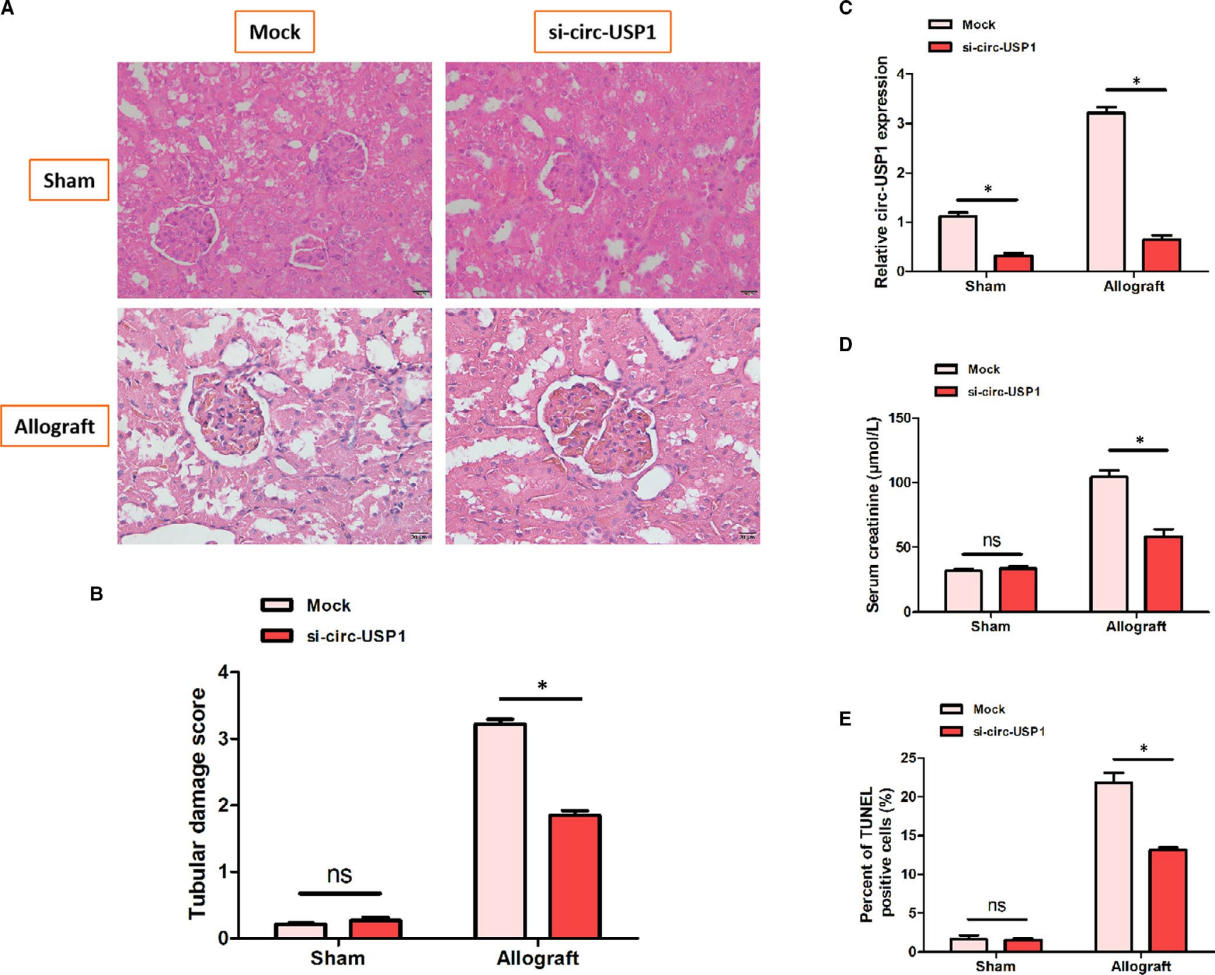
Silencing circ‐USP1 reduced injury in the kidney of mice that received renal allografts. A, Representative histological images of kidney tissues in the four groups. B, Tubular damage scores for kidney tissues in the four groups. C, Relative circ‐USP1 expression in kidney tissues in the four groups. D, Serum creatinine levels in the four groups. E, The ratio of TUNEL‐positive cells in the four groups. *P < .05

To examine the vital role of circ‐USP1 in mice renal allografts, the protein was delivered to the animals. The HE results and tubular damage scores showed that injury to the tubules significantly reduced after silencing circ‐USP1 (Figure 2A,B). Moreover, the expression of circ‐USP1 was remarkably down‐regulated in the si‐circ‐USP1 group compared with the mock group, as displayed in Figure 2C. Notably, no significant difference was observed in the serum creatinine levels and the percentage of TUNEL‐positive cells between the sham+mock and sham+si‐circ‐USP1 groups. However, the serum creatinine levels and the ratio of TUNEL‐positive cells were evidently lower in the allograft+si‐circ‐USP1 group compared with the allograft+mock group.

### 3.3 miR‐194‐5p was a target for circ‐USP1

**FIGURE 3:**
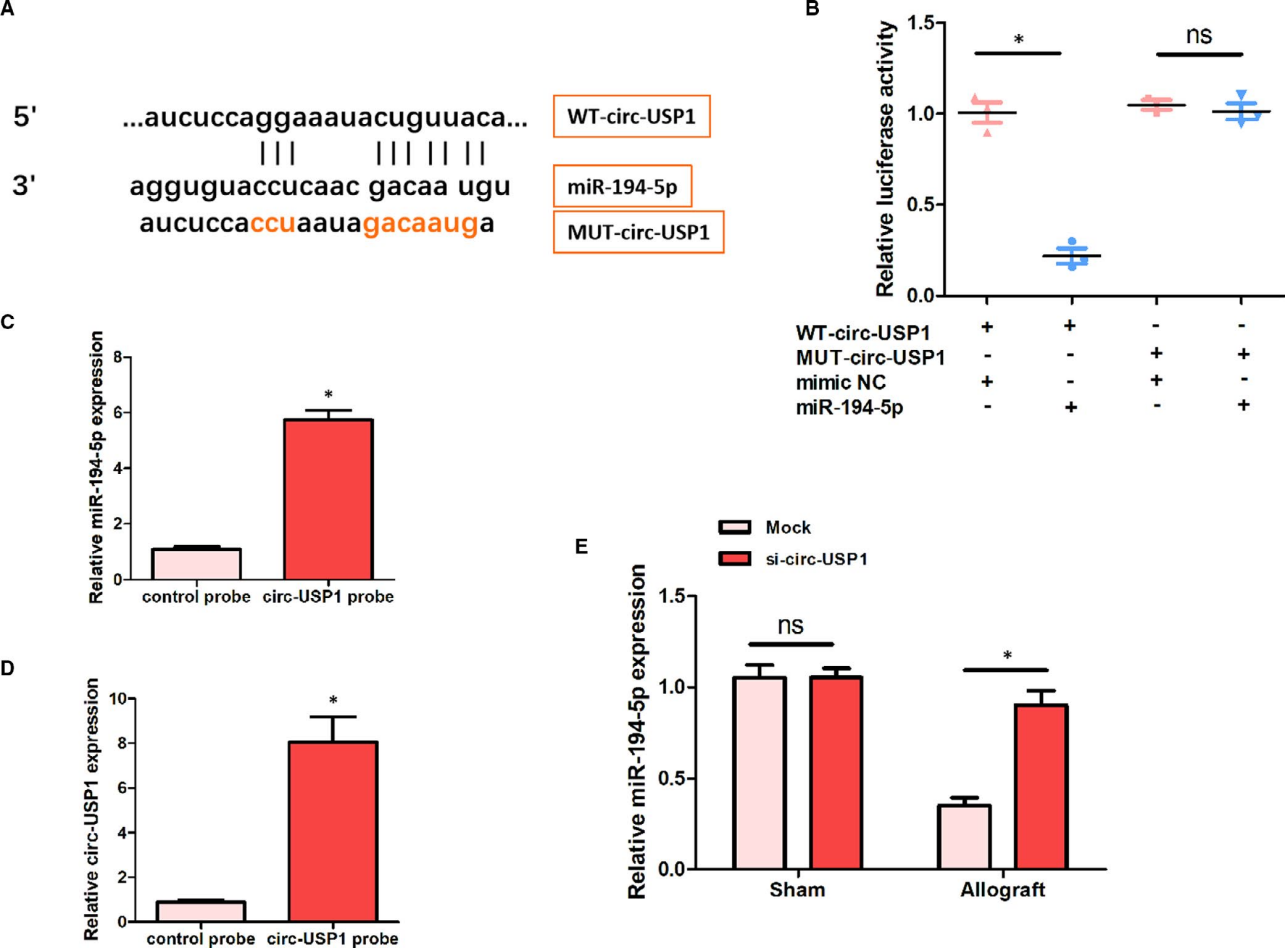
MiR‐194‐5p was a target for circ‐USP1. A, Schematic representation of binding sites between circ‐USP1 and miR‐194‐5p. B, Relative luciferase activities in HK‐2 cells in the four groups. C, Relative expression of miR‐194‐5p in HK‐2 cells in the two groups. D, Relative expression of circ‐USP1 in HK‐2 cells in the two groups. E, Relative expression of miR‐194‐5p in each group. *P < .05

Bioinformatics analysis showed that miR‐194‐5p might be a target for circ‐USP1, as can be seen from Figure 3A. Next, the interaction between miR‐194‐5p and circ‐USP1was examined through the luciferase reporter assay. Notably, luciferase activity was significantly reduced in the WT‐circ‐USP1+miR‐194‐5p group compared with the WT‐circ‐USP1+mimic NC group. However, there was no significant change in the luciferase activity of the MUT‐circ‐USP1 group, as outlined in Figure 3B. Compared with the control probe group, the expression of miR‐194‐5p and circ‐USP1 was significantly higher in the circ‐USP1 probe group in Figure 3C,D. Additionally, the over‐expression of circ‐USP1 remarkably reduced miR‐194‐5p expression in HK‐2 cells of the allograft group than that in the allograft+si‐circ‐USP1 group. Nonetheless, no significant change was witnessed in the expression of miR‐194‐5p between the sham+mock and sham+si‐circ‐USP1 groups (Figure 3E). These findings, therefore, reveal that miR‐194‐5p might be a target for circ‐USP1.

### 3.4 DNMT3A was a target of miR‐194‐5p

**FIGURE 4:**
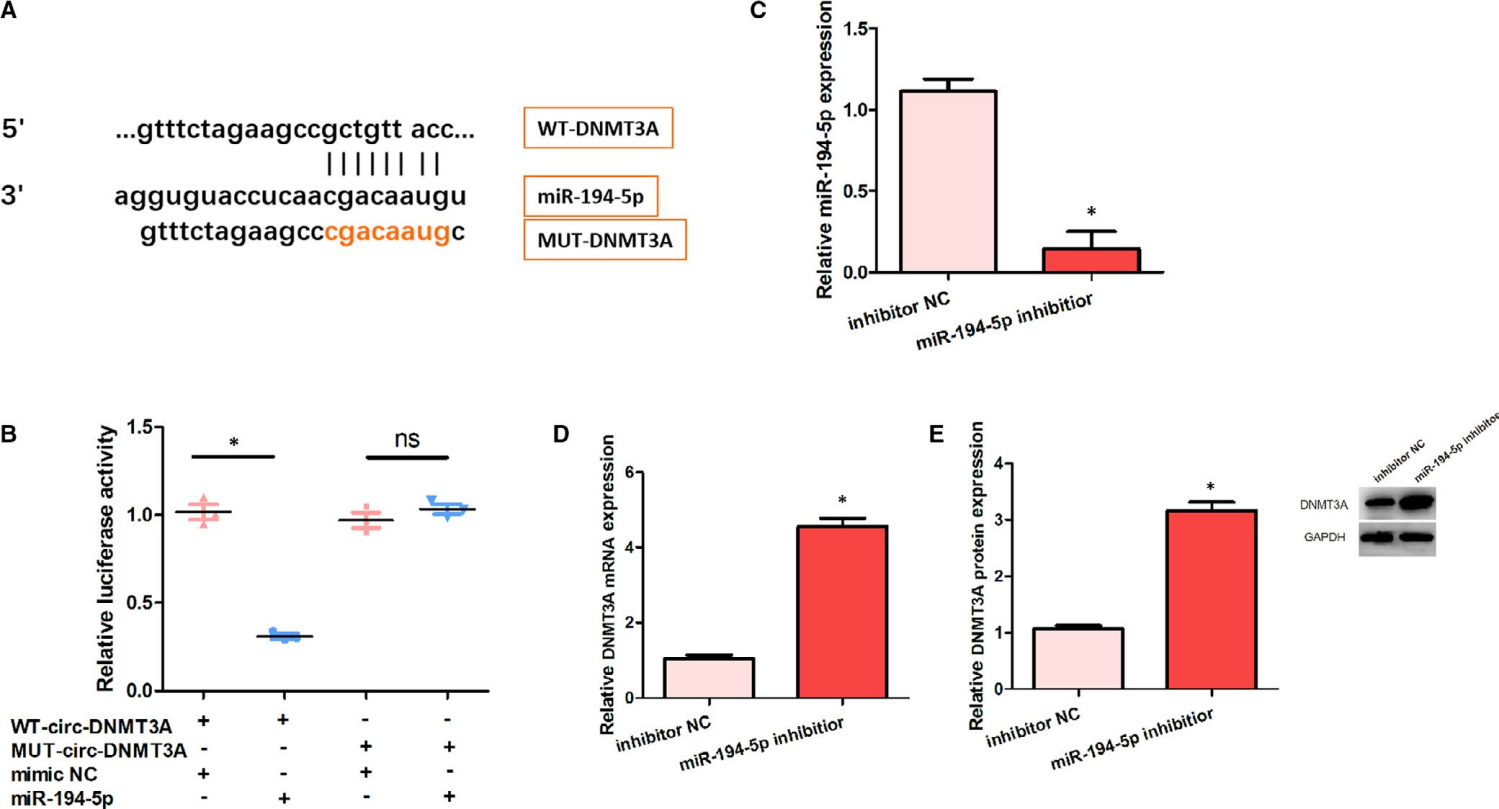
DNMT3A was a target of miR‐194‐5p. A, Schematic representation of binding sites between DNMT3A and miR‐194‐5p. B, Relative luciferase activities in HK‐2 cells in the four groups. C, Relative expression of miR‐194‐5p in HK‐2 cells in the two groups. D, Relative expression of DNMT3A mRNA in HK‐2 cells in the two groups. E, Relative expression of DNMT3A protein in HK‐2 cells in the two groups. *P < .05

Bioinformatic analysis indicated that DNMT3A might be a target of miR‐194‐5p (Figure 4A). Then, the correlation between DNMT3A and miR‐194‐5p was assessed by the luciferase reporter assay. The luciferase assay showed that miR‐194‐5p over‐expression decreased the activity of luciferase associated with WT‐circ‐DNMT3A but not the one related to MUT‐circ‐DNMT3A (Figure 4B). In addition, the expression of miR‐194‐5p was evidently lowered in the miR‐194‐5p inhibitor group than that in the inhibitor NC group (Figure 4C). Nevertheless, the relative DNMT3A mRNA and protein expression were significantly higher in the miR‐194‐5p inhibitor group, compared with the inhibitor NC group (Figure 4D,E). These data, therefore, reveal that DNMT3A was a target of miR‐194‐5p.

### 3.5 Silencing circ‐USP1 reduced expression of DNMT3A in the kidney of mice that received renal allografts

**FIGURE 5:**
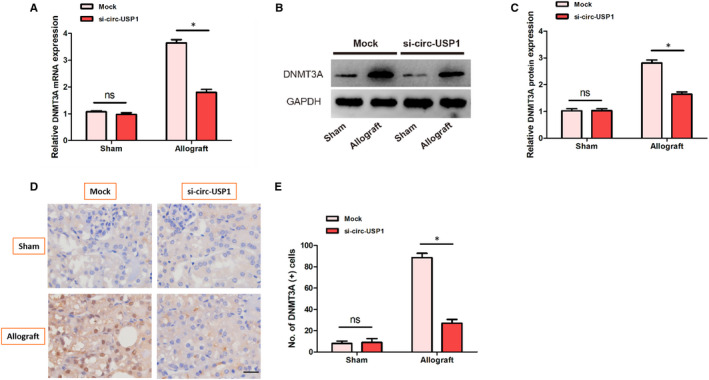
Silencing circ‐USP1 reduced DNMT3A expression in the kidney of mice that received renal allografts. A, Relative expression of the DNMT3A mRNA in four groups. B‐C, Relative expression of the DNMT3A protein in four groups. D‐E, Representative immunohistochemistry images of kidney tissues from sham and allograft mice. *P < .05

To further elucidate the association between DNMT3A and circ‐USP1, expression of DNMT3A was examined in the kidneys of mice. Western blot, qPCR and IHC results showed that DNMT3A was down‐regulated in allografts of the si‐circ‐USP1 group compared with the mock group as demonstrated in Figure 5A‐E.

### 3.6 Effects of circ‐USP1 and miR‐194‐5p on the expression of circ‐USP1, miR‐194‐5p and DNMT3A in H/R‐induced HK‐2 cells

**FIGURE 6:**
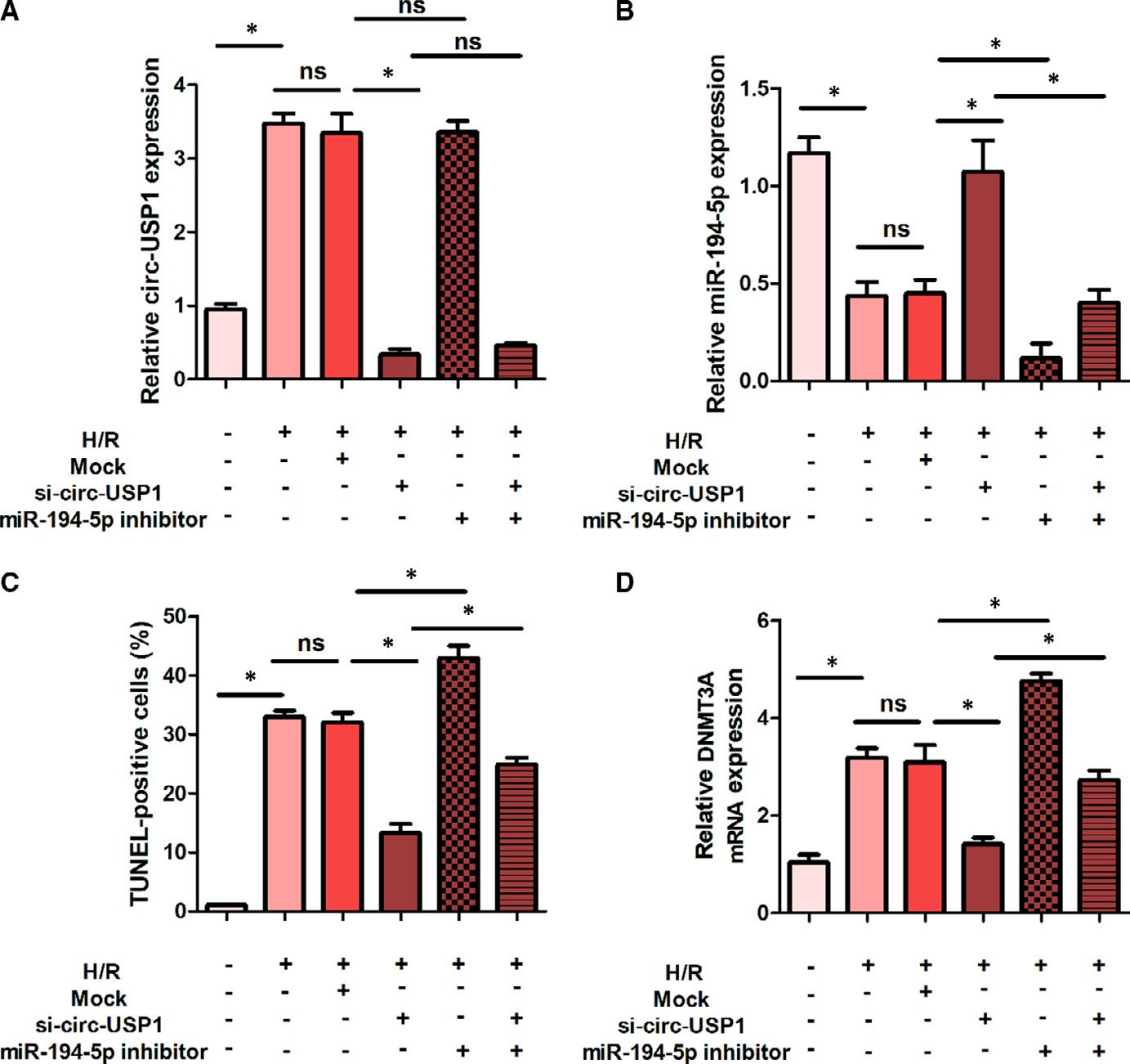
Effects of circ‐USP1 and miR‐194‐5p on the expression of circ‐USP1, miR‐194‐5p, and DNMT3A in H/R‐induced HK‐2 cells. A, Relative expression of circ‐USP1 in each group. B, Relative expression of miR‐194‐5p in each group. C, The rate of TUNEL‐positive cells in each group. D, Relative expression of DNMT3A in each group.*P < .05

As presented in Figure 6, compared with the control group, the expression of circ‐USP1, DNMT3A and the rate of TUNEL‐positive cells were significantly up‐regulated in the H/R group and the H/R+Mock group, while the expression of miR‐194‐5p was significantly down‐regulated. Additionally, compared with the H/R group and H/R+Mock group, the expression of circ‐USP1 and DNMT3A and the rate of TUNEL‐positive cells were remarkably decreased, while the expression of miR‐194‐5p was remarkably increased in the H/R+si‐circ‐USP1 group. DNMT3A expression and the rate of TUNEL‐positive cells were significantly up‐regulated in the H/R+miR‐194‐5p inhibitor group compared with the H/R+mock group, while the miR‐194‐5p expression was significantly decreased. Compared with the H/R+si‐circ‐USP1 group, DNMT3A expression and the rate of TUNEL‐positive cells in the H/R+si‐circ‐USP1+miR‐194‐5p inhibitor group were significantly increased, while the expression of miR‐194‐5p was significantly reduced.

## 4 DISCUSSION

Our study indicated that the expression of circ‐USP1 elevated, while that of miR‐194‐5p reduced in renal allografts in a time‐dependent manner. It was also shown that down‐regulation of circ‐USP1, through si‐circ‐USP1 delivery to mice, prevented kidneys from injuries in renal allografts. Additionally, lower circ‐USP1 expression was accompanied by higher miR‐194‐5p and lower DNMT3A expression in the kidney. This corroborates with previous reports showing that circRNAs may potentially be used as biomarkers or therapeutic targets for acute kidney injury.^24^

Although recent reports indicate that circ‐USP1 is involved in the dysregulation of endothelial cell functions,^19^ its role in kidney diseases remains largely unclear. Gao *et al* found that silencing circ‐USP increased permeability of the blood‐tumour barrier by decreasing tight junction‐related protein expression in cerebral microvascular endothelial cells in glioma.^19^ In this study, it was shown that silencing the circ‐USP1 protects renal tubule cells from apoptosis. Moreover, the study showed that miR‐194‐5p is a target for circ‐USP1. This finding is in line with that of Gao *et al*, showing that circ‐USP1 modulated the permeability of the blood‐tumour barrier through a miR‐194‐5p/FLI1‐mediated pathway.^19^ Another study indicated that lower expression of miR‐194‐5p and higher expression of MALAT1, ACVR2B were related with advanced TNM stage, larger tumour size (≥4 cm) and poor prognosis of patients with clear cell kidney carcinoma.^17^ Additionally, DNMT3A was demonstrated to target miR‐194‐5p and silencing circ‐USP1 reduced DNMT3A expression in the kidney of mice that had a renal allograft. This result is in agreement with a prior research presenting that down‐regulation of lncRNA NEAT1 modulated via miR‐194‐5p/DNMT3A enhanced the progression of acute myeloid leukaemia.^25^ Gondaliya et al^26^ found that miR29b might effectively target DNMT3B, DNMT1 and DNMT3A contributing in the development of diabetic nephropathy in renal proximal tubular cells.

Our study had some strengths. First of all, it was the first study to examine the role of circ‐USP1 in injuries caused by kidney transplantation. Secondly, it is the first research to explore the relationship between circ‐USP1, miR‐194‐5p and DNMT3A in kidney injury induced by hypoxia in acute renal allografts. In addition, our research group has also identified miR‐379‐3p and miR‐411‐3p as downstream targets of circ‐USP1, and we continue to conduct related experimental studies in the future.

In conclusion, circ‐USP1 functions as a competing endogenous RNA for miR‐194‐5p. This serves to regulate DNMT3A expression in kidney injury induced by hypoxia in acute renal allografts. Therefore, this finding indicates that circ‐USP1 may be considered as a vital therapeutic target in kidney injury.

## CONFLICT OF INTEREST

The authors confirm that there are no conflicts of interest.

## AUTHOR CONTRIBUTIONS

**Tao Yao:** Data curation (equal); Formal analysis (equal); Investigation (equal); Methodology (equal); Writing‐original draft (equal). **Dongqing Zha:** Data curation (equal); Methodology (equal); Resources (equal). **Ping Gao:** Data curation (supporting); Formal analysis (supporting); Software (equal). **Xiaoyan Wu:** Conceptualization (lead); Supervision (lead).

## Supporting information

Table S1

## Data Availability

Data are available on request from the authors.

## References

[1] Haberal M , Boyvat F , Akdur A , Kırnap M , Özçelik Ü , Yarbuğ KF . Surgical complications after kidney transplantation. Exp Clin Transplant. 2016;14:587‐595. 27934557

[2] Djamali A , Kaufman DB , Ellis TM , Zhong W , Matas A , Samaniego M . Diagnosis and management of antibody‐mediated rejection: current status and novel approaches. Am J Transplant. 2014;14:255‐271. 24401076 10.1111/ajt.12589 PMC4285166

[3] Sui W , Huang L , Dai Y , Chen J , Yan Q , Huang H . Proteomic profiling of renal allograft rejection in serum using magnetic bead‐based sample fractionation and MALDI‐TOF MS. Clin Exp Med. 2010;10:259‐268. 20376689 10.1007/s10238-010-0094-5

[4] Zheng J , Ding X , Tian X , et al. Assessment of different biomarkers provides valuable diagnostic standards in the evaluation of the risk of acute rejection. Acta Biochim Biophys Sin (Shanghai). 2012;44:730‐736. 22759804 10.1093/abbs/gms056

[5] Nair R , Agrawal N , Lebaeau M , Tuteja S , Chandran PKG , Suneja M . Late acute kidney transplant rejection: clinicopathological correlates and response to corticosteroid therapy. Transplant Proc. 2009;41:4150‐4153. 20005357 10.1016/j.transproceed.2009.09.074

[6] Rämisch S , Weininger U , Martinsson J , Akke M , André I . Computational design of a leucine‐rich repeat protein with a predefined geometry. Proc Natl Acad Sci USA. 2014;111:17875‐17880. 25427795 10.1073/pnas.1413638111 PMC4273356

[7] Meng X , Li X , Zhang P , Wang J , Zhou Y , Chen M . Circular RNA: an emerging key player in RNA world. Brief Bioinformatics. 2017;18:547‐557. 27255916 10.1093/bib/bbw045

[8] Liu J , Liu T , Wang X , He A . Circles reshaping the RNA world: from waste to treasure. Mol Cancer. 2017;16:58. 28279183 10.1186/s12943-017-0630-y PMC5345220

[9] Du WW , Fang L , Yang W , et al. Induction of tumor apoptosis through a circular RNA enhancing Foxo3 activity. Cell Death Differ. 2017;24:357‐370. 27886165 10.1038/cdd.2016.133 PMC5299715

[10] Kölling M , Haddad G , Wegmann U , et al. Circular RNAs in urine of kidney transplant patients with acute T cell‐mediated allograft rejection. Clin Chem. 2019;65:1287‐1294. 31371281 10.1373/clinchem.2019.305854

[11] Moore MJ , Scheel TKH , Luna JM , et al. miRNA‐target chimeras reveal miRNA 3'‐end pairing as a major determinant of Argonaute target specificity. Nat Commun. 2015;6:8864. 26602609 10.1038/ncomms9864 PMC4674787

[12] Bernardo BC , Ooi JYY , Lin RCY , McMullen JR . miRNA therapeutics: a new class of drugs with potential therapeutic applications in the heart. Future Med Chem. 2015;7:1771‐1792. 26399457 10.4155/fmc.15.107

[13] Choy JC . Granzymes and perforin in solid organ transplant rejection. Cell Death Differ. 2010;17:567‐576. 19876069 10.1038/cdd.2009.161

[14] Peng W , Chen J , Jiang Y , et al. Urinary fractalkine is a marker of acute rejection. Kidney Int. 2008;74:1454‐1460. 18800027 10.1038/ki.2008.459

[15] Anglicheau D , Suthanthiran M . Noninvasive prediction of organ graft rejection and outcome using gene expression patterns. Transplantation. 2008;86:192‐199. 18645476 10.1097/TP.0b013e31817eef7b PMC3595195

[16] Dell'Aversana C , Giorgio C , D'Amato L , et al. miR‐194‐5p/BCLAF1 deregulation in AML tumorigenesis. Leukemia. 2017;31:2315‐2325. 28216661 10.1038/leu.2017.64 PMC5668498

[17] Ye Y , Zhang F , Chen Q , Huang Z , Li M . LncRNA MALAT1 modified progression of clear cell kidney carcinoma (KIRC) by regulation of miR‐194‐5p/ACVR2B signaling. Mol Carcinog. 2019;58:279‐292. 30334578 10.1002/mc.22926

[18] Wang Y , Yang L , Chen T , et al. A novel lncRNA MCM3AP‐AS1 promotes the growth of hepatocellular carcinoma by targeting miR‐194‐5p/FOXA1 axis. Mol Cancer. 2019;18:28. 30782188 10.1186/s12943-019-0957-7 PMC6381672

[19] Gao Y , Wu P , Ma Y , et al. Circular RNA USP1 regulates the permeability of blood‐tumour barrier via miR‐194‐5p/FLI1 axis. J Cell Mol Med. 2020;24:342‐355. 31654502 10.1111/jcmm.14735 PMC6933377

[20] Brunetti L , Gundry MC , Goodell MA . DNMT3A in leukemia. Cold Spring Harb Perspect Med. 2017;7(2):a030320. 28003281 10.1101/cshperspect.a030320 PMC5287058

[21] Fang M , Liu S , Zhou Y , et al. Circular RNA involved in the protective effect of losartan on ischemia and reperfusion induced acute kidney injury in rat model. Am J Transl Res. 2019;11:1129‐1144. 30899412 PMC6413261

[22] Sonoda H , Lee BR , Park K‐H , et al. miRNA profiling of urinary exosomes to assess the progression of acute kidney injury. Sci Rep. 2019;9:4692. 30886169 10.1038/s41598-019-40747-8 PMC6423131

[23] Jablonski P , Howden BO , Rae DA , Birrell CS , Marshall VC , Tange J . An experimental model for assessment of renal recovery from warm ischemia. Transplantation. 1983;35:198‐204. 6340272 10.1097/00007890-198303000-00002

[24] Brandenburger T , Salgado Somoza A , Devaux Y , Lorenzen JM . Noncoding RNAs in acute kidney injury. Kidney Int. 2018;94:870‐881. 30348304 10.1016/j.kint.2018.06.033

[25] Duan M‐Y , Li M , Tian H , Tang G , Yang Y‐C , Peng N‐C . Down‐regulation of lncRNA NEAT1 regulated by miR‐194‐5p/DNMT3A facilitates acute myeloid leukemia. Blood Cells Mol Dis. 2020;82:102417. 32179410 10.1016/j.bcmd.2020.102417

[26] Gondaliya P , Dasare A , Srivastava A , Kalia K . miR29b regulates aberrant methylation in In‐Vitro diabetic nephropathy model of renal proximal tubular cells. PLoS ONE. 2018;13:e0208044. 30496316 10.1371/journal.pone.0208044 PMC6264835

