# Supplementary material for: Silencing circ‐USP1 protects the renal tubular from kidney injury induced by hypoxia via modulating miR‐194‐5p/DNMT3A axis in acute renal allografts
Source: J Cell Mol Med. 2021 Jan 23;25(13):5940–8. doi: 10.1111/jcmm.16286 (PMC8256350; doi:10.1111/jcmm.16286)
Supplement: Supplementary file 1 — Table S1 [file JCMM-25-5940-s001.docx]

**Table S1. All primers utilized in this study**

| **Primers** | |
| --- | --- |
| miR-194-5p (Forward primer) | CTAGTACCTAGAGGAACCTTTGAAGACTGTTACAGCTCAGCA |
| miR-194-5p (Reverse primer) | AGCTTGCTGAGCTGTAACAGTCTTCAAAGGTTCCTCTAGGTA |
| circ-USP1 (Forward primer) | GCGCCTAAGAGCTATTACCC |
| circ-USP1 (Reverse primer) | GTAGCCATT TTCCACTGCTC |
| DNMT3A (Forward primer) | GCGCCTCAGAGCTATTACCC |
| DNMT3A (Reverse primer) | GCAGCCATT TTCCACTGCTC |
| β-actin (Forward primer) | CGTTGACATCCGTAAAGACC |
| β-actin (Reverse primer) | AACAGTCCGCCTAGA AGCAC |
